# Physiological modules for generating discrete and rhythmic movements: component analysis of EMG signals

**DOI:** 10.3389/fncom.2014.00169

**Published:** 2015-01-09

**Authors:** Ana Bengoetxea, Françoise Leurs, Thomas Hoellinger, Ana Maria Cebolla, Bernard Dan, Guy Cheron, Joseph McIntyre

**Affiliations:** ^1^Laboratoire de Neurophysiologie et Biomécanique du Mouvement, Faculté des Sciences de la Motricité, Université Libre de BruxellesBrussels, Belgium; ^2^Departamento de Fisiología, Laboratorio de Cinesiología y Motricidad, Facultad de Medicina y Odontología, Universidad del País Vasco-Euskal Herriko Unibertsitatea (UPV/EHU)Leioa, Spain; ^3^Département de Neurologie, Hôpital Universitaire des Enfants Reine Fabiola, Université Libre de BruxellesBrussels, Belgium; ^4^Laboratoire d”Électrophysiologie, Université de Mons-HainautMons, Belgium; ^5^Heath Division, Fundacion Tecnalia Research and InnovationSan Sebastian, Spain; ^6^IKERBASQUE Science FoundationBilbao, Spain

**Keywords:** rhythmic movement, figure-eight, muscular synergy, principal component analysis, varimax factor analysis, upper limb

## Abstract

A central question in Neuroscience is that of how the nervous system generates the spatiotemporal commands needed to realize complex gestures, such as handwriting. A key postulate is that the central nervous system (CNS) builds up complex movements from a set of simpler motor primitives or control modules. In this study we examined the control modules underlying the generation of muscle activations when performing different types of movement: discrete, point-to-point movements in eight different directions and continuous figure-eight movements in both the normal, upright orientation and rotated 90°. To test for the effects of biomechanical constraints, movements were performed in the frontal-parallel or sagittal planes, corresponding to two different nominal flexion/abduction postures of the shoulder. In all cases we measured limb kinematics and surface electromyographic activity (EMG) signals for seven different muscles acting around the shoulder. We first performed principal component analysis (PCA) of the EMG signals on a movement-by-movement basis. We found a surprisingly consistent pattern of muscle groupings across movement types and movement planes, although we could detect systematic differences between the PCs derived from movements performed in each shoulder posture and between the principal components associated with the different orientations of the figure. Unexpectedly we found no systematic differences between the figure eights and the point-to-point movements. The first three principal components could be associated with a general co-contraction of all seven muscles plus two patterns of reciprocal activation. From these results, we surmise that both “discrete-rhythmic movements” such as the figure eight, and discrete point-to-point movement may be constructed from three different fundamental modules, one regulating the impedance of the limb over the time span of the movement and two others operating to generate movement, one aligned with the vertical and the other aligned with the horizontal.

## Introduction

Following the quantitative definitions for discrete and rhythmic gestures proposed by Hogan and Sternad (2007), handwriting movements, in terms of behavioral and observational features, are special cases of discrete movements because they have rhythmic phases but last a finite duration, with the hand starting and ending at zero velocity. Making the distinction between discrete and rhythmic movements is central because their underlying neural control could be different (Hollerbach, 1981). In fact, one can find in the literature three different proposals concerning the control of discrete vs. rhythmic movements. One view is that rhythmic movements are a concatenation of a series of discrete movements, the latter of which form the basic building blocks for complex movements (Abend et al., 1982; Soechting and Terzuolo, 1987a,b; Kalaska et al., 1997; Sabes, 2000). An opposing view is that rhythmic movements represent the fundamental class and that discrete movements are simply abbreviated rhythmic movements (Sternad and Schaal, 1999; Sternad et al., 2000; Schaal and Sternad, 2001; Sternad and Dean, 2003). Both of these viewpoints would suggest that only a single, common control mechanism is used to achieve both types of movement. A third possibility is that rhythmic and discrete movements represent two distinct movement classes that are mediated by separate neural control circuitry. Recent behavioral (Ikegami et al., 2010; Howard et al., 2011) and imaging studies (Schaal et al., 2004) support this latter hypothesis.

Numerous studies have examined the invariance of kinematic parameters for drawing movements, looking for the principles used by the central nervous system (CNS) for motor control (Viviani and Terzuolo, 1982; Lacquaniti et al., 1983; Viviani and McCollum, 1983; Soechting et al., 1986; Lacquaniti, 1989). Based on the kinematic invariance of the end-effector obtained, these authors have proposed that for curved movements the CNS respects the so-called “2/3 power law” and that each kinematic segment respects the same kinematic invariance presented by discrete movement, as specified by the “isochrony principle”. In light of these kinematic invariances, their conclusions have been used to support the hypothesis that the figure-eight and other “discrete-rhythmic” movements are composed of a series of concatenated discrete movements. Indeed, the observed presence of multiple peaks in the endpoint velocity profile might suggest that a figure-eight is composed of a series of superimposed discrete segments (Richardson and Flash, 2002). But kinematic segmentation doesn’t necessarily imply a segmented control of the movement (Sternad and Schaal, 1999). Indeed, evidence that the figure-eight is in fact an abbreviated rhythmic movement is emerging (Bengoetxea et al., 2010).

Within the set of all handwriting movements, the figure-eight is of particular interest from a theoretical and experimental point of view because it can be described as a Lissajous figure for which the vertical and horizontal frequency components are in an exact ratio of 2 (Buchanan et al., 1996). A figure eight can therefore also be described as the result of two coupled oscillators acting in perpendicular directions over a finite number of cycles (two horizontal cycles and one vertical cycle, to be exact). Although we previously demonstrated that for rapid execution of a single figure-eight movement the isochrony principle and the 2/3 power law between angular velocity and curvature are respected, and that the tangential velocity profile is invariant relative to the initial direction of movement (Cheron et al., 1999), electromyographic activity (EMG) analyses have shown that muscular activations present temporal modulation related to the figure as a whole, in contrast to directional pattern of tuning that would point to a segmented control. Moreover, we have shown that the prime movers are partitioned into two sets of synergistic muscles acting in a reciprocal mode and this reciprocal command was highly correlated with the spatial component of the velocity presenting the highest frequency (in the case of a vertical figure-eight the horizontal velocity component) (Bengoetxea et al., 2010). These results pointed to one or more oscillators controlling two muscular synergies.

In the study presented here we set out to determine if the modules underlying the production of discrete-rhythmic movements, in terms of muscle synergies, reflect an organization based on a series of discrete movements or on a combination of abbreviated oscillations. We reasoned that if two orthogonal coupled oscillators underlie the execution of the figure-eight movement, these oscillators should define two muscular synergies, each one dedicated to one of the two spatial components of the kinematics. On the other hand, we know that the synergistic organization is flexible and that a single muscle may be a member of more than one synergy (Tresch et al., 1999; Weiss and Flanders, 2004). We also know that EMG patterns are modulated by movement direction in 3D space (Flanders et al., 1994, 1996; Hoffman and Strick, 1999) and that muscle activation depend on its mechanical action, which depend on joint position (Hogan, 1985; Buneo et al., 1997). Finally, we know that the mapping of required muscle forces and joint torques is most often under constrained, allowing the CNS to exploit additional degrees-of-freedom to tune other properties of the musculoskeletal system, such as limb impedance (Hogan, 1985). We therefore looked at how each of these considerations influences the grouping of muscles into functional modules.

In the present work we asked how movement type (discrete vs. discrete-rhythmic), in addition to directional and biomechanical constraints, affects the organization of modules used to generate movements of the arm. We used principal component analysis (PCA) and varimax factor analysis to extract synchronous synergies (d’Avella and Bizzi, 2005; Klein Breteler et al., 2007) to see the relative involvement of each recorded muscle. We compared the synergies identified by these methods between different orientations, joint configurations and directions of movement for the figure eight and between figure eights and discrete point-to-point movements. In a companion article (see Bengoetxea et al., 2014) we combined this factor analysis with the identification of the relationship between EMG and movement parameters via a dynamic recurrent neuronal network (DRNN), in order to link the muscular synergies extracted with the movement generated. These two studies revealed a high-level of communality between the production of discrete and discrete-rhythmic movements and suggest an organization of motor control constructed from one or more modules controlling limb dynamical properties (e.g., impedance) and multiple modules that elicit reciprocal activation of opposing muscles to generate forces and movement.

## Material and methods

Data were collected from a total of 8 right-handed subjects, 4 males and 4 females, aged between 21 and 40 years. All were in good health, free from known neurological disorders, and had given informed consent to take part in the study, which was approved by the ethics committee at Brugman Hospital in Brussels (“Comité d’éthique hospitalier”—OM26). Data from one subject were unfortunately unusable due to a technical problem, leaving a total subject pool of 7 (3 males, 4 females).

Subjects were asked to draw, as fast as possible, figure-eight movements in free space with the right arm fully extended at the elbow (for more details see Bengoetxea et al., 2010). Movements were initiated in the center of the figure with an initial up-right (UR), down-right (DR), up-left (UL) or down-left (DL) direction with respect to external coordinates and subjects performed each of these movements twice. Two trials for one subject were lost for technical reasons, leaving a total of 7 × 4 × 2 − 2 = 56 figure-eight movements in the frontal plane. All seven subjects also performed eight point-to-point movements starting from a central target, one in each of eight different directions. In addition, three subjects (subjects 1, 2 and 3) performed figure-eight movements in both the frontal and sagittal workspaces, while the four other subjects (subjects 4, 5, 6 and 7) performed “horizontal” figure-eight movements (figure eights rotated in the frontal plane by 90°, such that the long axis of the figure was horizontal, instead of vertical). A part of the data has been reported in a previous study (data from the four subjects performing figure eights in the frontal and sagittal planes, see Bengoetxea et al., 2010). Data from the discrete movements performed by these subjects, and from the figure-eight and discrete movements from the three other subjects, are reported for the first time here and in our companion article in this issue.

### Data acquisition

Data acquisition methods were the same for both the previously reported data sets (Bengoetxea et al., 2010) and the new data reported here. Movements of the index finger were recorded and analyzed using the optoelectronic ELITE system (2 CCD-cameras, sampling rate of 100 Hz) (BTS, Milan) (Ferrigno and Pedotti, 1985). The cameras were placed 4 m apart from each other and 4 m from the subject. Four markers were attached to the arm (on the acromion, the lateral condoyle of the humerus, the radial apophysis of the wrist and the index finger). Velocity signals were obtained by digitally differentiating position signals using a fifth-order polynomial approximation. Reconstruction of the arm movements by the ELITE system using the trajectories of the 4 markers confirmed the visual observation that the upper arm, forearm, hand and index finger acted as a rigid link (Bengoetxea et al., 2010). Thus, we analyzed here only the index-finger marker that was used to trace the figure-eight.

Surface EMG was recorded with the TELEMG system (BTS, Milan) synchronized with the kinematic data. Silver-silver chloride electrode pairs (interelectrode distance of 2.5 cm) were placed over the belly of the following 7 muscles: posterior deltoid (PD), anterior deltoid (AD), median deltoid (MD), pectoralis major superior and inferior (PMS and PMI), latissimus dorsi (LD) and teres major (TM). Raw EMG signals (differential detection) were amplified by a portable unit 1000 times and transmitted to the main unit with a telemetry system (Telemg, BTS). A functional resistance test that isolated specific muscles was made in order to verify the absence of cross talk between adjacent muscles. Thereafter, EMGs were band-pass filtered (10–500 Hz), digitized at 1 kHz, full-wave rectified and smoothed by means of a third-order averaging filter with a time constant of 20 ms (Hof and Van den Berg, 1981).

### Component analysis

In the first part of our study we set out to identify synchronous synergies (d’Avella and Bizzi, 2005) using PCA. The input to the PCA was the EMG signal for each muscle and each figure-eight movement. The EMG signals were first normalized on a movement-by-movement basis for the discrete-rhythmic movements. For each EMG recording, the minimum value over the entire signal for each movement was subtracted and the maximum value was used to normalize the peak EMG signal during figure-eight movements. With this normalization, all EMG signals for each movement ranged from 0 and 1. A similar analysis was performed on a set of 8 point-to-point movements concatenated together, one in each of 8 directions, to produce the principal components associated with the production of discrete movements (Klein Breteler et al., 2007). We performed the PCA using the Statistica (© Statsoft) factor analysis module. This analysis resulted in 7 principle component vectors each composed of 7 loading factors (W1_muscle_–W7_muscle_) corresponding to the weights given to the EMG from each of the 7 muscles for each factor.

We focused the subsequent analysis of the principal component decompositions on the first 3 principal components, as these components accounted for 83.01 ± 2.84 % of the variance in the EMG data (mean across movements). We also computed the varimax rotation (Kaiser, 1958) of the first three principal components for each movement to generate a new set of three orthogonal loading vectors for each movement.

Because we computed the principal components on a movement-by-movement basis, we obtained multiple principal component and varimax decompositions for each direction, plane, figure-eight orientation and movement type. The principal component calculation, by design, assigns loading vectors in decreasing order according to the amount of variance explained by each one. If one adopts the basic premise that principal components reflect an underlying module or synergy, it is possible for a given synergy to be represented by the first, second or third principal component for a given movement trial, if the amount of movement (variance) associated with a given synergy increases or decreases between trials. We therefore used k-means clustering, with the number of clusters set to three, as an objective means to assign each loading vector to the group PC1, PC2 or PC3 based on similarity rather than on the amount of variance explained. The clustering algorithm was applied the set of first three principal component loadings collected across all movements, all mixed together for a total of 351 vectors, without regard for each vector’s ranking within the trial from which it was obtained. If the synergies are stable across movement types and subjects, one would expect that one of the three loading vectors from each movement would be assigned to PC1, one to PC2 and one to PC3. In the rare case where the k-means clustering assigned two loading vectors from a single movement trial to the same cluster, the loading vector with the highest distance from the cluster mean was shifted to the cluster that was left unassigned for that trial. A similar process was applied to assign the varimax loading vectors in each trial to groups VM1, VM2 and VM3.

The time courses of the activation of each principle component (PC1–PC3) and varimax loading (VM1–VM3) were then computed by projecting, at each time step, the vector of 7 muscle EMGs onto the loading vector describing each component. Note that the calculation of the covariance used to compute the PCA removes the mean from each of the 7 columns of the input matrix (i.e., removes the average EMG at the input) and also scales each input so that each channel has a variance equal to 1. The reconstructed EMG signals were therefore scaled and offset appropriately to account for this scaling of the inputs to the PCA.

#### Statistical analyses

We considered that the loading vectors associated to PC1, PC2 and PC3 within a given condition were sufficiently similar to allow the PCs to be compared based on the mean and variance of the loading vectors computed across subjects. This assertion is supported by two statistical arguments. First, we performed the k-means cluster analysis on the ensemble of loading vectors identified by PCA for the figure-eight movements performed in the frontal plane. Out of 54 movements (162 principal-component loading vectors), only one PC1 loading vector was misclassified into the cluster containing PC2s. Thus, the principal components were highly repeatable and unambiguously grouped into three clusters. We further verified that the loading values for each muscle and each PC across subjects did not violate the assumption of a normal distribution, according to the Kolmogorov-Smirnov (K-S) test (*p* > 0.20 in all cases). We therefore used MANOVA to compare the average PC1, PC2 or PC3 vectors across different conditions. Whenever the MANOVA revealed a significant difference (*p* < 0.01) of the loading vectors between conditions we performed a one-way ANOVA muscle-by-muscle to determine which muscle loadings were affected.

Loading vectors based on the varimax rotation (VM1, VM2 and VM3) were somewhat less distinct across trials. Using the same k-means clustering as described for the principal components in the frontal plane, there were more instances (8 out of 54 movements) where the k-means clustering attributed two loading vectors from the same movement to the same group. Furthermore, even after correcting these cases by reclassifying the vector with the largest distance from the cluster mean, the loading values for individual muscles did not always follow the normal distribution across trials (K-S: *p* < 0.05). Nevertheless, based on visual inspection and the central limit theorem, we considered that the within-subject averages could be compared across trials as a means of detecting systematic changes between conditions. Indeed, when we computed the average VM1, VM2 and VM3 for each subject across all 4 figure-eight movement directions (See Section Results for further details), the individual weight for each muscle of these average loading vectors did respect the normal distribution (K-S: *p* > 0.2). We therefore also applied MANOVA to compare VM1, VM2 and VM3 for different types of movement, as we did for the principal components PC1, PC2 and PC3.

To compare which of the two factoring methods (principal component or varimax) produced the least variation in loadings across subjects, we counted the number of times that the cluster analysis assigned two loading vectors from the same movement to the same cluster, with the underlying assumption that the more the loading vectors varied in terms of directions, the higher the chance that such misclassification can occur. We also computed the distance from the cluster mean for each loading vector and applied a one-way ANOVA with component (PC1, PC2, PC3, VM1, VM2, VM3) as the independent factor as a measure of the dispersion of individual vectors within each cluster.

## Results

We first looked to see if the PCA, which we applied to each movement one-by-one, was able to identify regular patterns of muscle involvement across the different movement directions and movement planes. Given that subjects may differ in the way that muscles may be organized into modules or synergies, we analyzed first the results from a single representative subject. This is the same subject whose data was used to train the artificial neural network in our companion study (see Bengoetxea et al., 2014). We then analyzed the principal components obtained across all participants to look for systematic, subject-independent changes in potential muscle synergies between conditions.

### PCA analysis

**Figure 1:**
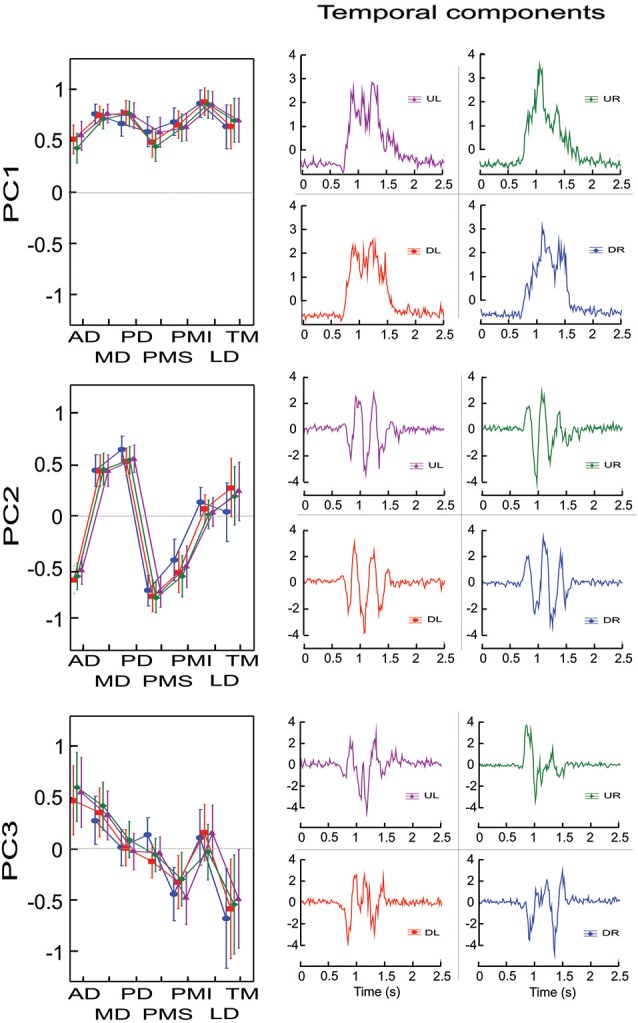
**Factor loadings and temporal components for un-rotated principal component analyses for one representative subject and for each initial direction movement**. The left column presents the mean factor loadings for PC1, PC2 and PC3 for each of the 7 muscles recorded. AD: anterior deltoid, MD: medial deltoid, PD: posterior deltoid, PMS: pectoralis major superior, PMI: pectoralis major inferior, LD: latissimus dorsi and TM: teres major. The right column present the temporal component for each PC and each direction. Each direction is identified by colors. UR: green, UL: purple, DR: blue and DL: red.

Figure 1 illustrates for the one representative subject the factor loadings for the 3 first principal components (left column) for each initial direction movement, the latter represented by different colors and symbols. From the factor loadings, one can observe that PC1 included a contribution of all 7 muscles in a synergistic pattern (all weights were positive), PC2 identified a reciprocal pattern of activation (positive and negative weights) between MD, PD and TM on one side and AD, PMS and PMI on the other (LD had loadings close to 0), while PC3 identified a different reciprocal relationship with AD and MD clearly on one side and PMI and TM clearly on the other (PD, PMS and LD had loadings close to 0). It is interesting to note that in PC2 and PC3 two groups of muscles appeared according to their mechanical actions. For PC2, the two sets of muscles have opposite actions with respect to horizontal (left-right) movements, while for PC3, the groups of muscles have opposite actions with respect to vertical (up-down) movements.

Figure 1 (right column) also shows the temporal evolution of the principal components for each of the 4 different movement directions (UL, UR, DL, DR). One can see that PC1 showed activation over the duration of the movement, with little or no activity in the stationary phase of the recording (prior to 0.5 s and after 2.0 s in the figure shown). There was little difference between the 4 different movement directions. The time course of the second and third PCs both showed significant modulation over the course of the movement that depended on the direction. PC2 presented 3 positive and 2 negative peaks for the UR and DR directions and 3 negative and 2 positive peaks for the UL and DL directions. PC3 also showed temporal modulation, but differed in terms of the number of peaks compared to PC2. Specifically, PC3 presented 1 negative and 2 positive peaks for the UL and UR directions and 1 positive and 2 negative peaks for the DL and DR directions.

To characterize to what extent the different muscles participated in each principal component, independent of their direction of action, we performed for this subject an ANOVA with muscle and PC as repeated measures on the absolute values of the loadings factors, with principal component (PC1, PC2, PC3) and muscle (AD, MD, PD, PMS, PMI, LD, TM) as independent factors. The cross-effect showed a significant difference between muscles and PC (*F*_(12,84)_ = 48.012, *p* < 0.001). Scheffe’s *post-hoc* analyses showed that AD and PMI participated with similar loadings in all three PCs (mean loading ± SD were 0.53 ± 0.1, 0.52 ± 0.11, 0.54 ± 0.1 for AD for PC1, PC2 and PC3 respectively and 0.65 ± 0.07, 0.50 ± 0.09, 0.39 ± 0.09 for PMI). MD and LD participated significantly more in the first PC compared to PC2 and PC3 (*p* < 0.03), whereas they showed little or no difference between PC2 and PC3. LD had loadings near to 0 for PC2 and PC3 while MD participated in both PC2 and PC3 at the same level as AD and PMI (mean loadings ± SD were 0.75 ± 0.03, 0.46 ± 0.05, 0.35 ± 0.05 for MD for PC1, PC2 and PC3 respectively and 0.86 ± 0.02, 0.1 ± 0.07 and 0.11 ± 0.07 for LD). PD and PMS had the same level of participation for PC1 and PC2 but were not implicate in PC3 (mean loadings ± SD were 0.74 ± 0.05, 0.59 ± 0.05 and 0.04 ± 0.03 for PD for PC1, PC2 and PC3 respectively and 0.53 ± 0.09, 0.75 ± 0.05 and 0.1 ± 0.07 for PMS). TM was the only recorded muscle that had the same level of activity in PC3 as in PC1 (0.58 ± 0.1, 0.67 ± 0.06) and participated lightly in PC2 (0.21 ± 0.12).

**Figure 2:**
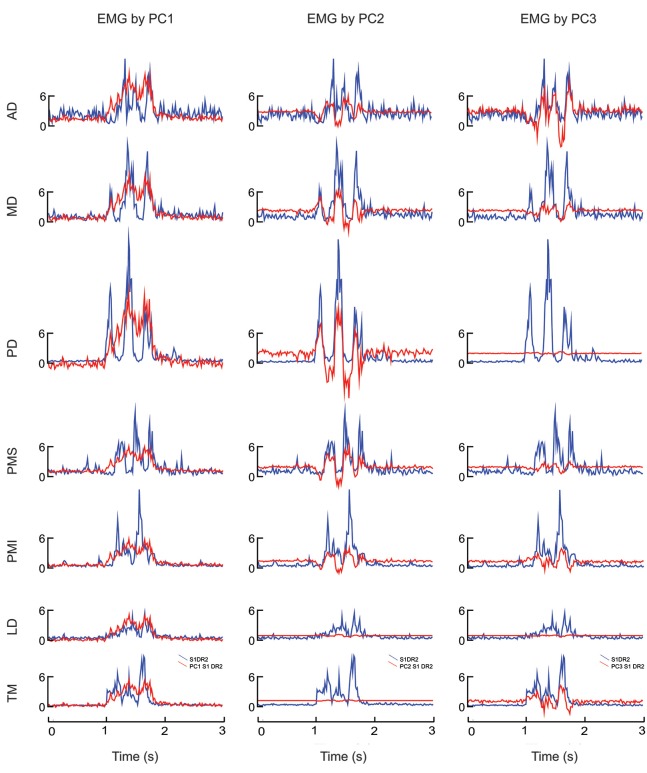
**EMG reconstructed by principal component analysis applied to one typical movement (figure-eight initiated in the down-right direction)**. Each column shows the EMG signal within each muscle associated with PC1 (left column), PC2 (middle column) and PC3 (right column). Blue traces show the actual smoothed EMG signal, whereas red shows the EMG signal reconstructed by the each weighted PC waveform. AD: anterior deltoid, MD: medial deltoid, PD: posterior deltoid, PMS: pectoralis major superior, PMI: pectoralis major inferior, LD: latissimus dorsi and TM: teres major.

Figure 2 illustrates the EMG signals for each muscle corresponding to the time course and loadings of each of the first 3 principal components, shown here for the movement initiated downward and to the right (DR). The reconstructed signals reinforce the interpretation given previously about the role of each principal component (synergy) in the execution of the movement. Specifically, one can observe a co-contraction of all muscles during the movement for EMG reconstructed from PC1, whereas synergies from PC2 and PC3 produced reciprocal activation patterns for which not all muscles participated at the same level. For EMG activations reconstructed from PC2 the figure illustrates that for a movement initiated in the down and right direction, MD and PD were the prime movers (for a right arm their action is to move the arm to the right) and presented a reciprocal command with respect to AD, PMS and PMI. LD and TM are not implicated in this synergy. The synergy extracted by PC3 shows that TM and PMI were agonists and presented the first activity given the fact that they are muscles that move the arm downward and presented a reciprocal activity with respect to AD, MD and PMS. But the reciprocal activity for PC3 was less “pure” than for PC2.

**Figure 3:**
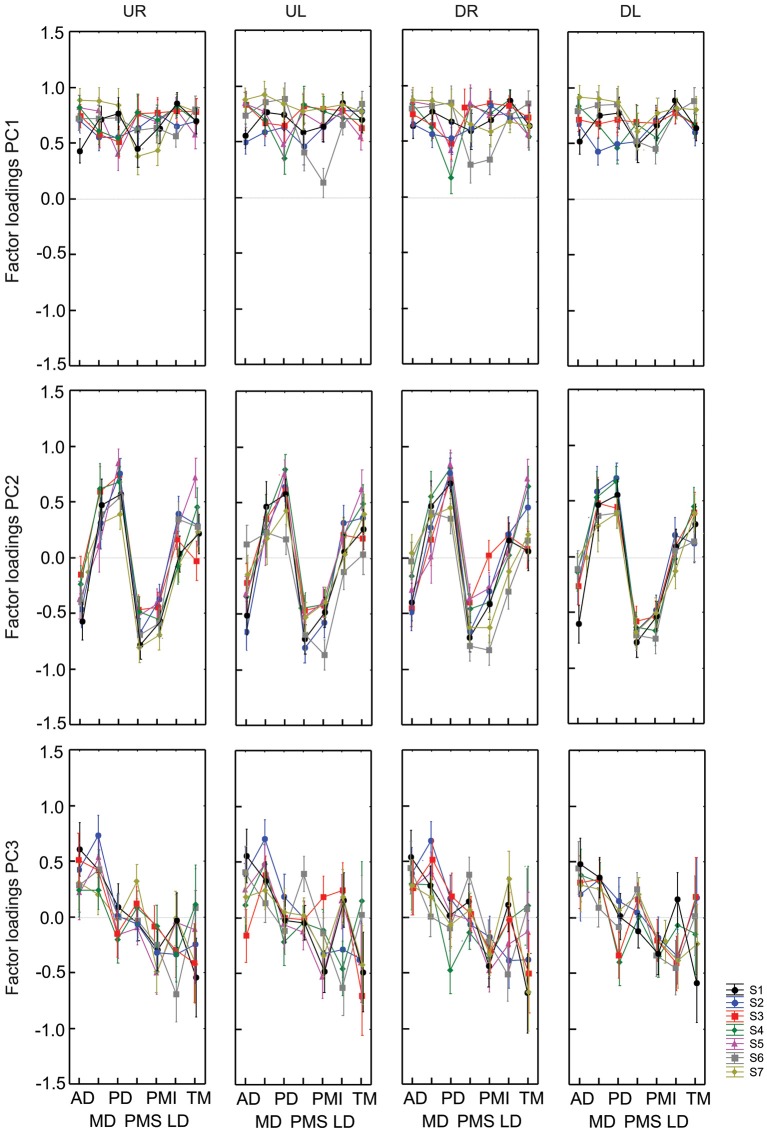
**Factor loadings (weighting) for PC1, PC2 and PC3 and each initial direction of vertical figure eights for all subjects**. Each graph represents factors loadings for each muscle for a given direction and PC. Results from each of 7 subjects are identified by colors and symbols. PC are organized by row while the direction of movements are displayed in columns.

Figure 3 shows the muscle loadings for each of the first three principal components for all subjects, separated as a function of PC and of movement direction. The loading factors were remarkably similar for the 7 different subjects; the average inter-subject standard deviation for each muscle and each factor was 0.133 ± 0.047. This observation, plus the stability in the cluster analysis of PCs (See Section Methods), justified the statistical comparison of loading patterns across subjects.

### Factors affecting muscle synergies

Based on the analysis and observations presented above we proceeded to analyze the data based on the means and variances across subjects of the muscle loadings for each of the first three principal components. We considered four main factors that might affect how muscles are grouped into synchronous synergies:

1. The temporal sequence of hand velocities (4 movement directions).
2. The frequency component of oscillations (vertical vs. horizontal figure-eight).
3. Anatomical constraints of different joint configurations (frontal vs. sagittal planes).
4. The type of movement (discrete vs. discrete-rhythmic).

**Figure 4:**
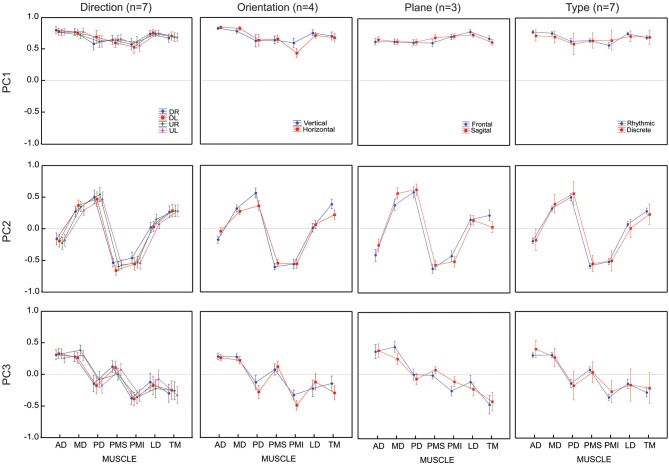
**Average loadings for each PC (un-rotated principal component analyses) and for 4 movement conditions (4 movement directions, 2 movement orientations, 2 movement planes and 2 movement types)**. Movement conditions are displayed in columns. Each PC is displayed in rows. Each graph presents the average loadings for each muscle for each PC and each condition (identified by color and symbols).

**Figure 5:**
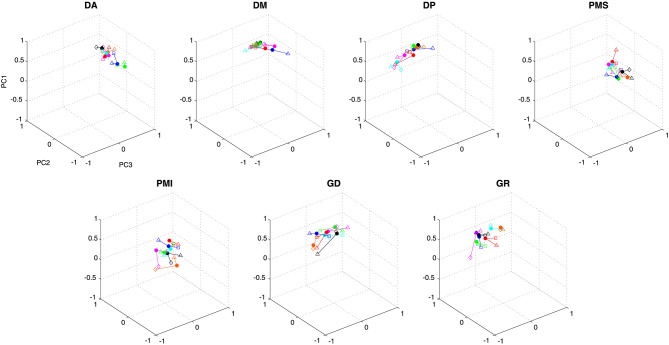
**Contribution of each muscle to the first three principal components. Individual data points show the average weighting for each subject in each condition**. Data from a single subject are grouped by color and by connecting lines, while symbols indicate the different conditions (filled circles—vertical figure eight in the frontal plane; triangles—discrete movements in the frontal plane; squares—horizontal figure eight in the frontal plane; diamonds—vertical figure eight in the sagittal plane).

The search for potential effects of factors 1 and 4 addressed the primary questions that motivated our study, i.e., how might time series of movement directions affect the grouping of muscles into modules or synergies? Thus, all seven subjects were asked to perform trials to allow these two contrasts. The other two factors (figure orientation and workspace) addressed secondary questions that provided interesting benchmarks with which to compare the primary results. For practical reasons, therefore, we asked only 4 of our subject to perform figure eights in both the vertical and horizontal directions and only 3 of our subject to perform movements in both the frontal and sagittal planes. The results of each of these contrasts are described below. The loading vectors averaged across subjects are shown in Figure 4 while Figure 5 shows the contribution of each muscle to each of the first three principal components for each subject and each condition. The details of the statistical tests are reported in Table 1.

**Table 1:** **Statistics for raw PCA**.

MANOVA				AD	MD	PD	PMS	PMI	LD	TM
*Movement direction (UR-UL-DR-DL)*
PC1	Wilks *λ* = 0.8466	*F*_(21,218.78)_ = 0.6217	*p* = 0.9005
PC2	Wilks *λ* = 0.7315	*F*_(21,218.78)_ = 1.1894	*p* = 0.2622
PC3	Wilks *λ* = 0.8430	*F*_(21,218.78)_ = 0.6387	*p* = 0.8872
*Figure-eight orientation (vertical vs. horizontal)*
PC1	Wilks *λ* = 0.6519	*F*_(7,54)_ = 4.1194	*p* = 0.0011					**
PC2	Wilks *λ* = 0.5619	*F*_(7,54)_ = 6.0145	*p* < 0.0001	**		**				**
PC3	Wilks *λ* = 0.6138	*F*_(7,54)_ = 4.8536	*p* = 0.0003			*		*		*
*Movement plane (frontal vs. sagittal)*
PC1	Wilks *λ* = 0.6769	*F*_(7,40)_ = 2.7271	*p* = 0.0206				**
PC2	Wilks *λ* = 0.5863	*F*_(7,40)_ = 4.0328	*p* = 0.0020	**	**					**
PC3	Wilks *λ* = 0.6083	*F*_(7,40)_ = 3.6797	*p* = 0.0037		**			*
*Type of movement (discrete-rhythmic vs. discrete)*
PC1	Wilks *λ* = 0.9456	*F*_(7,85)_ = 0.6981	*p* = 0.6735
PC2	Wilks *λ* = 0.9678	*F*_(7,85)_ = 0.4037	*p* = 0.8976
PC3	Wilks *λ* = 0.9224	*F*_(7,85)_ = 1.0222	*p* = 0.4217

Significant differences identified by post hoc analyses (right columns) are indicated by * (p < 0.05) and ** (p < 0.01).

#### Movement direction

All seven subjects performed the figure-eight movement two times in each of the four possible directions. The loadings within a given PC were remarkably similar across the four different movement directions. There was no significant difference between the loading vectors computed across subjects for each direction (*p* > 0.2, see Table 1) and one-way ANOVA test conducted separately on each muscle loading for each PC confirmed that the loadings assigned to each muscle did not change for any of the first three PCs as a function of movement direction.

#### Figure-eight orientation

Four subjects traced out figure eights in both the vertical and horizontal orientations in the frontal plane. The average loading vectors across subjects were qualitatively very similar for the two orientations. Nevertheless, some reliable differences were detected. The MANOVA test of loading vectors was significant for each of the three PCs (*p* < 0.01). One-way ANOVAs computed *post-hoc* showed that PD, PMI and TM all had significantly greater weight (more negative values) in PC3 for the horizontal vs. vertical orientations. There was a concomitant decrease in the weight of PMI in PC1 and of PD and TM in PC2. AD had somewhat less influence in PC2 (less negative weight) in the horizontal figure eight, but there was no change in AD”s contribution to either PC1 or PC3.

#### Frontal vs. sagittal plane

Three subjects performed the figure eight movements in two different nominal orientations of the outstretched arm: with the arm extended straight ahead (frontal plane) and with the arm stretched out straight to the side (sagittal plane). Again, the loadings for each PC were qualitatively similar between the two conditions, but with some small variations. The MANOVA showed only a marginally significant difference between the planes for PC1 (*p* = 0.0206) but statistically reliable difference between the planes for PC2 and PC3 (*p* < 0.01). One-way ANOVAs applied *post hoc* demonstrated that PMS had a slightly greater influence in PC1, MD had a greater influence and AD and TM had lesser influences on PC2 and MD and PMI had less weight in PC3, in the sagittal compared to the frontal plane of movement.

#### Discrete vs. discrete-rhythmic movements

All seven subjects performed both the figure eights and the point-to-point movements in the frontal plane. As for the comparison between movement directions, there was no apparent difference in the principal components computed for the discrete-rhythmic figure eights and the discrete point-to-point movements. The MANOVA showed no significant difference (*p* > 0.4) between movement types for any of the three principal components PC1, PC2 and PC3.

### Varimax rotation

**Figure 6:**
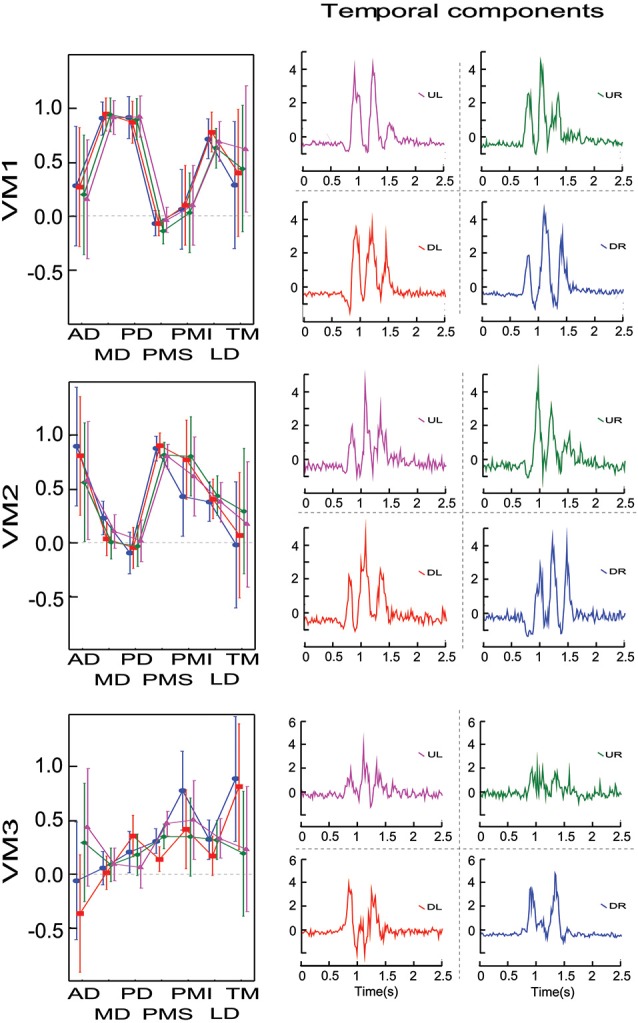
**Factor loadings and time course for the varimax components for one representative subject and for each initial direction movement**. Left column presents the mean factor loadings for VM1, VM2 and VM3 for each of the 7 muscles recorded. Right column presents the temporal component for each varimax component and each direction.

Compared to the un-rotated principal components computed for S1 (Figure 1), the varimax rotation for the same subject (Figure 6) grouped muscles into components (synergies) in a quite different fashion. Rather than identifying a “co-activation” module and two “reciprocal” modules, as seen for the first three principle components, the three varimax components could be described as one that drives more-or-less rightward rotations (MD, PD, LD), one that drives more or less leftward rotations (AD, PMS, PMI), and one that favors muscles with a component of action in the downward direction for movements of the outstretched arm (PMI, LD, TM). But this is a gross over-simplification and the participation of the different muscles in the three varimax loading vectors, which was much more mixed in terms of direction of action. Indeed, even if VM1 is dominated by muscles that rotate the outstretched arm rightward (MD, PD), other muscles (LD, TM) participate just as much in VM1 as in VM3. Similarly, PD contributes as much to VM3 as it does to VM1.

**Figure 7:**
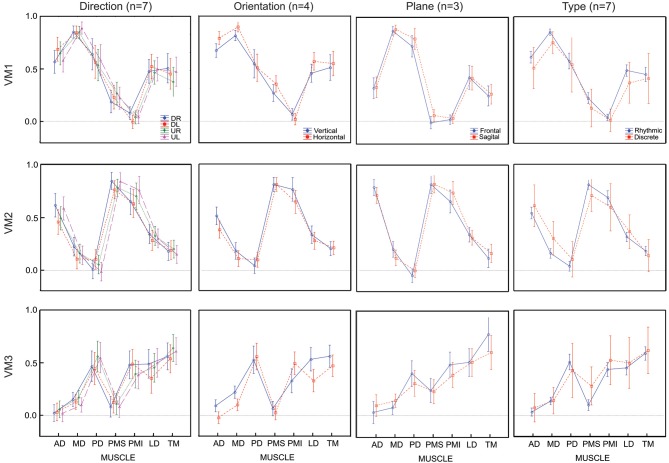
**Average loadings for each varimax component (VM) and for 4 movement conditions (4 movement directions, 2 movement orientations, 2 movement planes and 2 movement types)**. Movement conditions are displayed in columns, varimax components are displayed by rows. Each graph presents the average loadings for each muscle for each PC and each condition identified by color and symbols.

**Figure 8:**
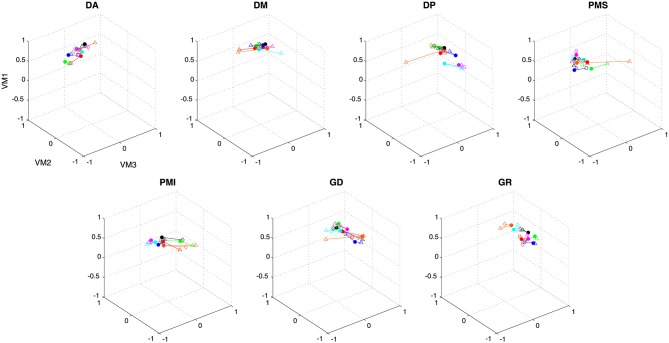
**Contribution of each muscle to the first three varimax components. Individual data points show the average weighting for each subject in each condition**. Data from a single subject are grouped by color and by connecting lines, while symbols indicate the different conditions (see caption, Figure 5).

Figure 7 shows the comparison of average loading for VM1, VM2 and VM3 as a function of movement direction (UR, UL, DR, DL), figure-eight orientation (horizontal, vertical), movement plane (frontal or sagittal) and movement type (discrete-rhythmic or discrete). Figure 8 shows the contribution of each muscle to each varimax component, computed separately for each subject and each condition. The only highly reliable difference found in the MANOVA analysis of these data (see Table 2) was in the comparison between the vertical and horizontal figure-eight orientations (*p* < 0.01 for VM1 and VM3; *p* = 0.056 for VM2). *Post-hoc* analyses showed fewer and statistically weaker differences (*p* < 0.05) in the loading for individual muscles, compared to the equivalent tests applied to the principal components.

**Table 2:** **Statistics for varimax rotation**.

MANOVA				AD	MD	PD	PMS	PMI	LD	TM
*Movement direction (UR-UL-DR-DL)*
VM1	Wilks *λ* = 0.7944	*F*_(21,218.78)_ = 0.8696	*p* = 0.6306
VM2	Wilks *λ* = 0.7591	*F*_(21,218.78)_ = 1.0496	*p* = 0.4054
VM3	Wilks *λ* = 0.8193	*F*_(21,218.78)_ = 0.7487	*p* = 0.8872
*Figure-eight orientation (vertical vs. horizontal)*
VM1	Wilks *λ* = 0.6273	*F*_(7,54)_ = 4.5827	*p* = 0.0005
VM2	Wilks *λ* = 0.7835	*F*_(7,54)_ = 2.1315	*p* = 0.0554	*				*
VM3	Wilks *λ* = 0.51346	*F*_(7,54)_ = 7.3098	*p* < 0.0001					*	*
*Movement plane (frontal vs. sagittal)*
VM1	Wilks *λ* = 0.6781	*F*_(7,40)_ = 2.7125	*p* = 0.0212
VM2	Wilks *λ* = 0.8170	*F*_(7,40)_ = 1.2802	*p* = 0.2847
VM3	Wilks *λ* = 0.7374	*F*_(7,40)_ = 2.0355	*p* = 0.0742
*Type of movement (discrete-rhythmic vs. discrete)*
VM1	Wilks *λ* = 0.9077	*F*_(7,85)_ = 1.2351	*p* = 0.2928
VM2	Wilks *λ* = 0.9402	*F*_(7,85)_ = 0.7729	*p* = 0.6116
VM3	Wilks *λ* = 0.9292	*F*_(7,85)_ = 0.9256	*p* = 0.4911

Significant differences identified by post hoc analyses (right columns) are indicated by * (p < 0.05).

### Comparing PCA vs. varimax

By mathematic principle, the varimax rotation of the three first principal components explains the same amount of variance in the data as the first three principal components do themselves. So one cannot say that one decomposition is to be preferred over the other on that basis. We instead asked whether the PCA or varimax decomposition was more invariant across subjects and conditions. Two observations suggest that the first three principal components were somewhat more regular than the corresponding varimax rotation. First, in the k-means clustering process that we used to assign the PCA vectors to the PC1, PC2 or PC3 groups and to assign the varimax vectors to the VM1, VM2 and VM3 groups, one would expect that if the three orthogonal vectors are aligned across subjects and conditions, the k-means algorithm should assign one vector from each individual decomposition to each group. If the orientation of the three vectors varies significantly, however, from the mean, a given vector might fall between two clusters, causing two vectors from the same decomposition to be assigned to the same group. This happened 3 times for the principal components and 21 times for the varimax decompositions, out of a total of 117 movements. Second, an ANOVA applied to the distance from the mean for each component cluster (PC1, PC2, PC3, VM1, VM2, VM3) showed a significant main effect (*F*_(5,696)_ = 50.69, *p* < 0.001) and a planned comparison showed a significant difference overall between the principal component clusters and the varimax clusters (*F*_(1,696)_ = 36.09, *p* < 0.001). Scheffe’s *post-hoc* analysis showed the PC1 had the least average distance; PC2 and VM2 were next, followed by PC3, VM1 and VM3. Thus, overall the principal component decomposition was less variable across movements, with the difference being mainly attributed to the lower inter-trial variability of the co-activation component defined by PC1.

## Discussion

In this study we looked for modularity in patterns of muscle activation used to perform discrete-rhythmic movements, a class of movements typical of handwriting (Hogan and Sternad, 2007), and we compared the underlying structure with that identified for discrete movements performed in eight different directions in the frontal plane. We asked subjects to draw figure eights in different directions, in different orientations and in two different nominal arm postures. Thus, in addition to the main comparison between discrete and discrete-rhythmic movements, we also considered how the underlying modules might be tuned as a function of the directional, biomechanical and rhythmic constraints.

PCA, and other forms of factor analysis, have in recent years become an important tool to identify the muscular synergies underlying human movement, from reaching (d’Avella et al., 2003; Bizzi et al., 2008) to locomotion (Ivanenko et al., 2004; Dominici et al., 2011) passing through complex movements (Weiss and Flanders, 2004; Klein Breteler et al., 2007; Danna-Dos-Santos et al., 2008). Here we used PCA and the varimax rotation as means to identify structure in the activation of different muscles.

Both the three principal component vectors and the three varimax vectors were remarkably stable across the different movement directions, figure-eight orientations, joint configurations (movement plane) and the types of movement (discrete or rhythmic). There were small, but measurable differences in loading between the figure-eight orientations (horizontal or vertical) and between the movement’s planes (frontal or sagittal). It should be noted, however, that fewer subjects performed the movements in each of these two conditions, whereas as all 7 subjects performed the figure eights and discrete movements in the frontal plane. It is possible that the orientation and movement-plane comparisons were more sensitive to inter-individual changes between conditions due to the lower N in each case. Furthermore, we do not exclude the possibility that loadings change between movement directions and movement types for individual subjects. But the main result, in the context of the questions evoked in the Introduction, is that overall the synchronous synergies, whether identified through PCA or varimax rotation, were no more affected by the type of movement (rhythmic or discrete) than by changing the time series of movement directions, the organization of oscillations in cyclic movements or the biomechanical constraints. This observation runs counter to our hypothesis by which we expected the CNS to exploit the redundant degrees of freedom within the system to select synergies that would be best adapted to the performance of one or the other type of movement. Our component analyses suggest that three main modules can be extracted for the movements described here, because they capture the bulk of the variation in EMG signals. For the un-rotated principal components, the first component showed a general co-activation of all the muscles, irrespective of the type of movement or the initial direction. This co-activation started and ended with the movement despite the fact that before and after the movement the arm was held in a static position. The co-contraction induced by PC1 would tend to stiffen the arm and thus serve to stabilize the arm’s posture before, during and after the movement and to tune the impedance of the limb to meet the demands of the movements to follow. The second and the third principal components each showed a pattern of reciprocal activation but differed in terms of how the muscles were grouped. Whereas the second module encompassed muscles that are antagonistic in terms of their horizontal direction of action, the same muscles were divided in the third module according to their vertical direction of action. Under this decomposition, the actual movement would then be realized by two reciprocal synergies represented by PC2 and PC3. According to the parlance proposed by Hogan and Sternad (2012), PC1 would constitute a “mechanical impedance” synergy while PC2 and PC3 would each be representative of “oscillation” synergies. Such a decomposition would be particularly adapted to rhythmic movements were each reciprocal synergy could be associated with a separate oscillator.

In contrast, each of the varimax components manifested only non-negative weightings (on average). The varimax decomposition would be consistent with “sub-movement” synergies that would tend to push the limb in one direction or another through the activation of a set of agonistic muscles, without the automatic co-activation or reciprocal de-activation of the effective antagonists (Hogan and Sternad, 2012). The varimax decomposition is more representative of a vector strategy in which each underlying module drives the limb in a given direction, reflecting the fact that muscle can pull, but not push, and thus cannot be negatively active. Modulation of limb impedance can, nevertheless, be achieved through the varimax decomposition, even if there is no identified co-activation module *per se*. Co-activation, and thus impedance modulation, could be achieved by recruiting simultaneously VM1, VM2 and VM3, while cyclic movements could be achieved by various activations of the same modules to generate movement in different directions.

### Methodology

One might ask to what extent the details of the analysis procedures play a role in the modules that we observed. For instance, it is known that PCA are potentially sensitive to the normalizations applied to the input data. In this study we set out to compute the principal components on a movement-by-movement basis, thus allowing us to examine the stability of the principal component decompositions across repeated movements in the same conditions and across different conditions by using standard statistical methods such as ANOVA.^1^ But the algorithms for PCA transform the incoming data to be centered on zero with variance equal to one, essentially normalizing the data on a trial-by-trial basis. By doing so, we open up the possibility that factor decompositions might change from one movement to the next due to the scaling factors that also changed from trial to trial. Surprisingly, our data showed that the decompositions were very stable, despite potential variability stemming from the normalization procedure. Our trial-by-trial normalization represents the more conservative method vis-à-vis our conclusions that synchronous muscular synergies vary little between discrete and discrete-rhythmic movements.

A second, perhaps more fundamental question is that of the factorization methods used to analyze the data. Different approaches of factor analysis have been used in the past to extract synergies, and the results obtained depend on the method used (Tresch et al., 2006). Here we compared the results from two different methods, varimax vs. unrotated principal components. Can one claim that the varimax is a better description of the underlying neural structure than the un-rotated principal components, or vice versa, based on our empirical observations? Both the un-rotated principal components and the varimax decompositions are mathematically valid solutions that describe equally well the variance of the various EMG signals. We therefore asked whether one or the other provided a more consistent representation of muscle activation patterns across subjects and across movements. In our conditions we found that the principal component decomposition was less variable than the varimax decomposition when computed on a trial-by-trial basis. One might expect to see such a result if the neural hardware indeed organizes muscles into a fixed set of synergies according to PC1, PC2 and PC3. Thus, these observations support the hypothesis that muscles are organized in a set of co-contraction and reciprocal synergies. These observations do not, however, constitute a definitive proof, due to properties of the principal component computation. Principal component vectors are in fact the eigenvectors of a covariance matrix. Those vectors are distinct and well defined when the eigenvalues corresponding to each vector are different. Had the first and second principal components accounted for similar amounts of variance in the EMG, the directions of the first and second PCs would be ill-defined and one would expect them to vary considerably just due to measurement noise. By analogy, the process of finding the varimax solution might add variability across trials if the optimal solution is not sharply defined in each case.

By considering the un-rotated principal components and the varimax rotation of the same data we have therefore evoked an interesting contrast in the way that movements can be generated through the action of muscles. We note, however, that this clear contrast between the two identification strategies was a fortuitous outcome of our experimental conditions. The varimax rotation that we used here does not explicitly seek to generate only positive loading factors for muscles; it just happened to do so for the movement studied here. Recent studies (Ivanenko et al., 2004; Tresch et al., 2006; d’Avella et al., 2008; Delis et al., 2014) have employed the technique of non-negative factorization to explicitly find such solutions. Similarly, PCA does not explicitly seek to group muscles into a co-contraction module plus reciprocal activation, but that also happened to be the outcome of the analysis of our data. Future studies could use instead a factorization algorithm that explicitly looks to organize components in this manner. For instance, a hierarchical factor analysis could be use, where the “secondary” factor would identify the co-contraction unit while a rotation of the primary vectors to maximize “reciprocity” could provide an appropriate solution for future studies.

The comparison of the two factorial decompositions presented here and the discussion above should therefore serve as a cautionary tale for future studies. From the purely mathematical analysis presented here, one cannot claim with high confidence that we have identified the neural structure of modules or synergies based only on the correlations between muscle activations. As we have shown here, the grouping of muscles into purported synergies through component analysis of EMG will depend highly on the *a priori* choice as to what type of factor analysis is performed and on the experimental conditions. Additional information is needed before one can state a clear preference for one decomposition over another. In our companion article, we endeavored to do just that, by using an artificial dynamic recurrent neural network to search for the relationship between EMG and movement. Nevertheless, the simple fact that 3 components can account for a large part of the variance in EMG signals, regardless of which rotation is used, is consistent with what one would predict if activation patterns are organized into synergies as a means of reducing the number of degrees of freedom in the mapping from desired movement to muscle activations.

### Implications for neural mechanisms

One can see in these analyses that, whichever decomposition is considered (PCA or varimax) two muscles might be agonistic in one synergy and antagonistic in another. It would be difficult to understand how the same muscle, if activated as a whole, could participate properly in both synergies. If we refer to the preferred action direction of motor units of the deltoid muscle (Herrmann and Flanders, 1998), most of the motor units exhibit a cosine tuning function showing a unique preferred direction. Thus, whereas a single muscle may be shared across muscle synergies, this sharing may be realized by incorporating the motor units of a given muscle into each synergy according to the preferred direction of each motor unit.

Transitions from fast discrete movements to rhythmic movement (Sternad et al., 2013) as well as transitions from slowly continuous movement to sub-movements (Teeken et al., 1996; van der Wel et al., 2009) reveal that the underlying controls for discrete and rhythmic movements are based on the same modules. Our results provide further evidence that muscular synergies underlying both types of movement are the same. Despite the fact that at the cerebral level the control of discrete vs. rhythmic movements has been shown to implicate different cortical areas (Schaal et al., 2004), the fact that directional discrete movements and rhythmic figure-eight present no differences in the three first principal components identified at the muscular level supports the hypothesis that discrete and rhythmic movements present the same neural control (Sternad et al., 2000, 2002; Sternad and Dean, 2003), at least at the level of synchronous muscular synergies.

The organization of those modules might, however, reflect higher levels of processing as well. In the case of our principal component decomposition, control would be shared between a co-contraction module and two reciprocal modules, the latter of which were surprisingly well aligned with the vertical and horizontal directions of movement (see also our companion paper for further evidence of this point). It is likely not a coincidence that the modules are oriented along these two canonical directions. One might hypothesize that the horizontal/vertical orientations of PC2 and PC3 are linked to the spatial characteristics of the figure eight, which was intrinsically orientated with the horizontal and vertical. But the PCA of our discrete movements was carried out separately from the computations on the figure eights, yet we found the same groupings in either case. Since the eight movement directions were equally spaced in all directions in the frontal plane, bias in the directions of movement cannot explain this phenomenon. Gravity itself acting on the arm could provide an explanation, as one might argue that the up/down synergies could take advantage of gravity as a driving force, reducing the amplitude of EMG modulation needed to produce movement in the vertical direction. But our EMG signals were normalized muscle-by-muscle, removing this as a possible explanation as well. On the other hand, there is ample evidence that human perception and visuomotor coordination is preferentially tuned to the vertical dimension tied to a multi-modal reference frame that includes the body axis and gravity (Howard, 1982; Paillard, 1991; Gentaz et al., 2001; McIntyre and Lipshits, 2008; Tagliabue and McIntyre, 2012). The organization of muscular synergies, which may be implemented at the level of the periphery, might nevertheless be tuned based on constraints defined in supraespinal areas involved with the processing of spatial information (Bizzi and Cheung, 2013).

## Conclusions

In this study we set out to compare discrete and discrete-rhythmic movements, in terms of synchronous muscular synergies than can be identified through principal component and varimax factor analysis. To this question we found a remarkably clear result: the invariance of the synchronous synergies, be they identified by principal components or varimax factors. This result suggests that a common mechanism underlies both types of movements, at least in terms of purported synergies that underlie the generation of muscle activation patterns. It is perhaps somewhat surprising that the CNSc does not exploit the additional degrees of freedom for generating forces to tune the system differently for these two classes of movements.

The secondary question of whether the principal components or varimax decompositions better represent the modules use to produce a certain class of upper-limb movements remains open. The un-rotated principal components suggested an organization based on a co-contraction module plus two modules for reciprocal activation, one horizontal and the other vertical. The varimax decomposition indicated instead a set of three basis vectors used to construct forces in different directions. Based on the analyses presented here, we argue for the co-contraction plus reciprocal organization, because of the somewhat less variability in the principal component decomposition and on conceptual grounds. Nevertheless, the arguments presented here are admittedly not conclusive. In our companion article we search for further evidence to support our hypothesis by using an artificial neural network to identify the functional significance, in terms of movement, of the modules identified here.

## Conflict of interest statement

The authors declare that the research was conducted in the absence of any commercial or financial relationships that could be construed as a potential conflict of interest.

## Footnotes

^1^However, that the discrete movements were normalized as a set (i.e., the EMG from each muscle was divided by its maximum value across all 8 movements) and the principal component analysis was applied to each set individually. This makes sense in that through the comparison of these data with the figure eights we were testing the null hypothesis (which actually proved to be true) that figure eight movements are constructed as a serial concatenation of modules used to produce discrete movements.
